# Supplementary material for: Dynalign II: common secondary structure prediction for RNA homologs with domain insertions
Source: Nucleic Acids Res. 2014 Nov 21;42(22):13939–48. doi: 10.1093/nar/gku1172 (PMC4267632; doi:10.1093/nar/gku1172)
Supplement: SUPPLEMENTARY DATA [file supp_gku1172_nar-02021-z-2014-File012.zip › manual/Text/partition.html]

RNAstructure Command Line Help: partition


|  |  |  |
| --- | --- | --- |
|  | RNAstructure Command Line Help  partition and partition-smp | - Contents - Index |
| partition is used to calculate the partition function for a sequence, which can be used to predict base pair probabilities. Note that the partition function save file remembers the nucleic acid type it was built with (RNA or DNA), so any further calculations with that file will assume they are done with that same nucleic acid type. partition-smp is a parallel processing version for use on multi-core computers, built using OpenMP. USAGE: partition <seq file> <pfs file> [options] OR: partition-smp <seq file> <pfs file> [options] Required parameters: <seq file> \| The name of a sequence file containing input data. Note that lowercase nucleotides are forced single-stranded in structure prediction.; <pfs file> \| The name of a binary partition function save file to which output will be written. Options which don't require added values: -d, -D, --DNA \| Specify that the sequence is DNA, and DNA parameters are to be used. Default is to use RNA parameters.; -h, -H, --help \| Display the usage details message. Options which require added values: -c, -C, --constraint \| Specify a folding constraints file to be applied. For partition, current supported constraints are force pairs, force a nucleotide to be single stranded, and force a nucleotide to be double stranded. Default is to have no constraints applied.; -dso, -DSO, --doubleOffset \| Specify a double-stranded offset file, which adds energy bonuses to particular double-stranded nucleotides. Default is to have no double-stranded offset specified.; -md, -MD, --maxdistance \| Specify a maximum pairing distance; that is, the maximum number of bases between the two nucleotides in a pair. Default is no restriction on the distance between pairs.; -sh, -SH, --SHAPE \| Specify a SHAPE data file to be used to generate restraints. These restraints specificially use SHAPE pseudoenergy restraints. Default is no SHAPE data file specified.; -si, -SI, --SHAPEintercept \| Specify an intercept used with SHAPE restraints. Default is -0.6 kcal/mol.; -sm, -SM, --SHAPEslope \| Specify a slope used with SHAPE restraints. Default is 1.8 kcal/mol.; -t, -T, --temperature \| Specify the temperature at which calculation takes place in Kelvin. Default is 310.15 K, which is 37 degrees C.; -x, -X, --experimentalPairBonus \| Specify a text file with a two-dimensional matrix of bonuses (in kcal/mol) to apply to each residue pair, as might be obtained from a mutate/map measurement. Matrix must have the same number of rows and columns as the target RNA. Bonus is applied once to edge base pairs, twice to internal base pairs. Default is no experimentalPairBonus file specified.; -xo \| Specify an intercept (overall offset) to use with the 2D experimental pairb onus file. Default is 0.0 (no change to input bonuses).; -xs \| Specify a number to multiply the 2D experimental pair bonus matrix. Default is 1.0 (no change to input bonuses). Notes for smp: partition-smp, by default, will use all available compute cores for processing. The number of cores used can be controlled by setting the OMP\_NUM\_THREADS environment variable. References 1. Reuter, J.S. and Mathews, D.H. "RNAstructure: software for RNA secondary structure prediction and analysis." *BMC Bioinformatics*, 11:129. (2010). 2. Mathews, D.H. "Using an RNA secondary structure partition function to determine confidence in base pairs predicted by free energy minimization." *RNA*, 10:1178-1190. (2004). 3. McCaskill, J.S. "The equilibrium partition function and base pair probabilities for RNA secondary structure." *Biopolymers*, 29:1105-1119. (1990). | | |
| Visit The Mathews Lab RNAstructure Page for updates and latest information. | | |
